# Harley Institute

**Published:** 1913-12-20

**Authors:** 


### "Harley Institute."

### "REFUSAL TO RENEW THE LICENCE."

Under this heading last week we reported fully the proceedings before the Public Control Committee of the London County Council on December 5, when the committee after deliberation refused to renew the employment agency licence to this institute.

Mr. S. A. Flemmer writes to ask us to point out that "no training school of massage holds a licence from the London County Council, and for that reason it cannot be refused."

No one who read the official report we published last week can be under any misapprehension on this point, for the report made it clear that the proceedings related to the employment agency licence.

